# Are artificial intelligence-generated recommendations clinically safe? Evaluation of thromboembolic prophylaxis advice after arthroplasty

**DOI:** 10.1590/1806-9282.20260626

**Published:** 2026-09-21

**Authors:** Ömer Polat, Alper Dünki, Mehmet Talha Aydin, Savaş Çamur

**Affiliations:** 1 University of Health Sciences, Umraniye Education and Research Hospital, Department of Orthopaedics and Traumatology – Istanbul, Türkiye

**Keywords:** Artificial intelligence, Arthroplasty, Venous thromboembolism, Decision support systems, clinical, Clinical decision-making

## Abstract

**OBJECTIVE:**

Venous thromboembolism prophylaxis remains a complex and controversial issue in orthopedic practice, with persistent variability in recommendations even among experts. With the growing use of online platforms and artificial intelligence, the aim of this study was to evaluate the quality and clinical adequacy of artificial intelligence-generated recommendations regarding venous thromboembolism.

**METHODS:**

Thirty-three guideline-based questions derived from International Consensus Meeting, National Institute for Health and Care Excellence, American College of Chest Physicians, and American Academy of Orthopaedic Surgeons recommendations were independently submitted to ChatGPT (Generative Pre-trained Transformer [GPT]-4.0) by two orthopedic surgeons, resulting in 66 generated responses for analysis. Responses were assessed using the DISCERN instrument, Journal of the American Medical Association benchmark criteria, and a novel clinically oriented scale, the Thromboembolism Digital Knowledge Level. Inter-rater reliability was evaluated using intraclass correlation coefficients.

**RESULTS:**

According to DISCERN, 62.1% of responses were classified as good quality, whereas Thromboembolism Digital Knowledge Level evaluation demonstrated that only 45.45% of responses were clinically adequate, with no responses classified as excellent. The same responses were systematically categorized into higher quality levels by DISCERN compared to Thromboembolism Digital Knowledge Level. All scoring systems demonstrated excellent inter-rater reliability (Intraclass Correlation Coefficient range: 0.897–0.961). Although differences between scoring systems were statistically significant, the effect size was moderate (r=0.39). Strong correlations were observed between evaluators across all scales (p<0.001).

**CONCLUSION:**

ChatGPT-generated responses demonstrated good structural quality but limited clinical adequacy for thromboembolic prophylaxis after arthroplasty. These findings suggest that artificial intelligence systems should be used cautiously and may not be sufficient as standalone clinical decision-support tools.

## INTRODUCTION

Prior to the routine implementation of venous thromboembolism (VTE) prophylaxis, the incidence of symptomatic VTE in patients requiring prolonged hospitalization ranged between 15 and 30%^1^. Following the introduction of prophylactic strategies, this rate has been reported to decrease to approximately 1.9–2% within 90 days^1^, and further reductions to as low as 0.3% have been achieved with advancements in perioperative management^2^. Achieving these outcomes requires adherence to established clinical guidelines. Among the most widely ­recognized are those published by the American College of Chest Physicians (ACCP), the American Academy of Orthopaedic Surgeons (AAOS), and the National Institute for Health and Care Excellence (NICE)^1,3,4^.

Although these guidelines provide comprehensive ­recommendations across various clinical scenarios, a universal consensus has not been fully established. To address this, the International Consensus Meeting (ICM), involving nearly 600 experts from multiple specialties, was convened to evaluate the existing literature and provide recommendations on VTE prophylaxis following orthopedic procedures^5,6^. Nevertheless, even recent efforts have not resulted in complete agreement across all clinical aspects.

Given that this treatment protocol remains a source of considerable ambiguity even among experts, we sought to examine how the internet and, more recently, widely adopted artificial intelligence (AI) systems provide recommendations ­regarding VTE. The rapid adoption of Chat Generative Pre-Trained Transformer (ChatGPT; OpenAI, San Francisco, CA, USA) by both the general public and healthcare professionals raises an important question: how reliable is the information it provides in a clinical context? ChatGPT is a large language model trained on extensive datasets and designed to generate contextually appropriate responses to user queries^7,8^.

Previous studies have primarily focused on the structural quality of AI-generated medical information, with ­limited evaluation of clinical accuracy and patient safety. While instruments such as DISCERN and Journal of the American Medical Association (JAMA) benchmarks assess the quality and transparency of information presentation, they do not directly evaluate clinical appropriateness or safety. Therefore, there is a need for clinically oriented assessment tools. In this study, we evaluated the medical adequacy and reliability of ChatGPT-generated responses to guideline-based questions on thromboembolic prophylaxis following arthroplasty. We hypothesized that ChatGPT would provide up-to-date but potentially less evidence-based recommendations.

## METHODS

This study did not involve human or animal participants, and therefore, institutional review board approval was not required due to the publicly accessible nature of ChatGPT.

A total of 33 guideline-based questions derived from ICM, NICE, ACCP, and AAOS recommendations were independently submitted to ChatGPT (Generative Pre-trained Transformer [GPT]-4.0) by two orthopedic surgeons, resulting in 66 ­generated responses for analysis. Each question was entered in a new chat session using identical standardized prompts and default system settings, and all responses were recorded without modification to ensure methodological reproducibility.

The collected responses were evaluated by the physicians using the DISCERN instrument and the JAMA benchmark criteria. DISCERN is a validated tool consisting of 16 questions across three domains, designed to assess the quality of written health information, with higher scores indicating better quality^9,10^. Scores were categorized as follows: 63–75 (excellent), 51–62 (good), 39–50 (moderate), 27–38 (poor), and 16–26 (very poor).

The JAMA benchmark criteria assess four domains: authorship, attribution, disclosure, and currency. Each domain is scored as 1 (present) or 0 (absent), with a total score ranging from 0 to 4^11^. While DISCERN and JAMA evaluate the structural quality and transparency of information, they do not directly assess clinical accuracy or patient safety.

To address this limitation, we developed a novel instrument, the Thromboembolism Digital Knowledge Level (TDKL) scale. The TDKL scale consists of 12 items across four domains: clarity and comprehensibility, therapeutic accuracy and clinical appropriateness, safety considerations, and scientific rationale (Table 1). Each item is scored from 0 to 2, yielding a total score ranging from 0 to 24, with higher scores indicating greater clinical adequacy.

**Table 1:** Thromboembolism Digital Knowledge Level scale.

Thromboembolısm dıgıtal knowledge level scale				
A. Clarity and comprehensibility (six points)		0	1	2
	1. The response is written in a clear, concise, and easily understandable manner.			
	2. The response directly addresses the question and provides relevant information.			
	3. The response maintains scientific clarity without unnecessary or unrelated details.			
B. Therapeutic information and clinical appropriateness (eight points)				
	4. The recommended treatment or clinical approach is scientifically accurate and up-to-date.			
	5. Treatment details are adequately described (e.g., dosage, duration, indications).			
	6. Alternative treatment options or management strategies are appropriately mentioned.			
	7. Additional protocols or considerations for high-risk groups or special clinical scenarios are provided when relevant.			
C. Safety information and complications (four points)				
	8. Potential complications related to the recommended treatment are correctly identified.			
	9. Preventive measures or management strategies for possible complications are included.			
D. Scientific rationale and evidence use (six points)				
	10. The response includes reference to scientific evidence or guideline-based rationale when appropriate.			
	11. The scientific information used (if present) is accurate, reliable, and up-to-date.			
	12. The interpretation of scientific data is correct and free from misrepresentation.			

Content validity of the TDKL scale was evaluated by eight orthopedic surgeons with expertise in thromboembolism. Each item was rated independently using a four-point Likert scale (1=not appropriate, 2=needs revision, 3=appropriate, 4=highly appropriate). Content validity and internal consistency of the TDKL scale were confirmed.

Statistical analyses were performed using Statistical Package for the Social Sciences software (version 27; IBM Corp., Armonk, NY, USA). Continuous variables were expressed as mean±standard deviation. During the scale development phase, ICC (2, k) was used to assess the reliability of mean ratings across multiple evaluators. In the primary analyses, ICC (2,1) was used to evaluate the consistency of individual raters. The relationship between the surgeons’ scores was assessed using Spearman's rank correlation analysis. Differences between scale scores were analyzed using the Wilcoxon signed-rank test. Correlation analyses were also performed for the TDKL subdimensions. A p-value of<0.05 was considered statistically significant.

## RESULTS

This study represents a cross-sectional analysis evaluating the medical adequacy of responses generated by an AI-based language model to guideline-based clinical questions on VTE.

**Figure 1:**
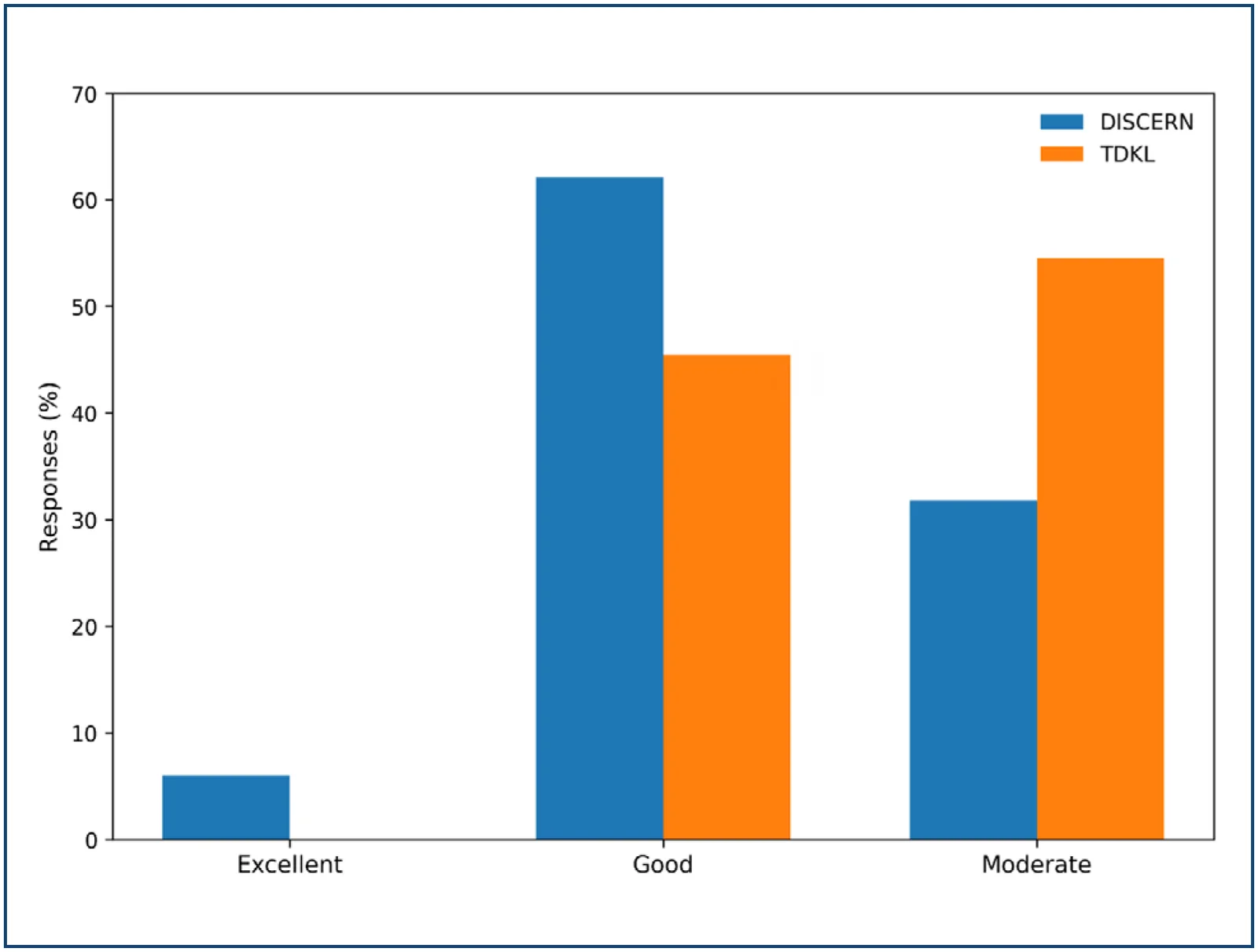
Descriptives of the results.

According to DISCERN scores, four responses (6%) were classified as excellent, 41 (62.1%) as good, and 21 (31.8%) as moderate. In contrast, based on TDKL scoring, 30 responses (45.45%) were classified as good, 36 (54.55%) as moderate, and none as excellent (Figure 1).

When DISCERN and TDKL classifications were compared, the same responses were found to be systematically categorized into different quality levels. Specifically, responses rated as "good" in terms of structural quality were frequently classified as "moderate" in terms of clinical adequacy.

All three evaluation tools demonstrated excellent inter-rater reliability. Although the TDKL scale showed the highest ICC values, overlapping confidence intervals precluded a definitive statistical superiority among the scales. Based on ICC (2,1) analysis, the TDKL scale demonstrated high agreement, indicating consistent evaluation of AI-generated responses across raters.

Despite statistically significant differences between scale scores, the effect size was moderate (r=0.39), suggesting that the observed differences, although significant, may have limited clinical impact.

Analysis of the relationship between the two evaluators’ scores revealed strong and statistically significant positive correlations across all scales (p<0.001), indicating a high level of scoring consistency (Table 2).

**Table 2:** Correlation analysis of DISCERN, JAMA, and Thromboembolism Digital Knowledge Level scores between two evaluators (Spearman's rho).

	Evaluator 1 (Mean±SD)	Evaluator 2 (Mean±SD)	p	rho
DISCERN	53.5±5.87	53.7±6.49	<0.001	0.885
JAMA	0.4±0.52	0.5±0.50	<0.001	0.942
TDKL	14.5±2.15	14.4±2.18	<0.001	0.964

p-values were calculated using Spearman's correlation analysis. TDKL: Thromboembolism Digital Knowledge Level.

## DISCUSSION

In this study, the medical adequacy of guideline-based responses generated by an AI system for thromboembolic prophylaxis was evaluated using DISCERN, JAMA benchmark criteria, and the newly developed TDKL scale.

The findings of this study indicate that although AI-generated responses are generally well structured and consistent across repeated queries, they may exhibit important limitations in terms of clinical adequacy and patient safety. While the majority of responses were categorized as "good quality" according to DISCERN, only approximately 40–45% were considered clinically adequate based on TDKL scoring. This discrepancy highlights the fundamental difference between structural quality and true clinical reliability. This discrepancy likely reflects the different domains evaluated by structural quality tools and clinically oriented assessment instruments.

Previous studies comparing AI-generated responses with expert consensus statements have demonstrated that AI systems tend to provide responses aligned with higher levels of evidence, yet may fail to incorporate experiential clinical judgment in scenarios with limited or ambiguous evidence^12^. However, the absence of references and limited transparency in AI-generated responses contribute to low JAMA benchmark scores. This finding reflects the inherent limitations of such models in terms of authorship, attribution, and currency, which are critical for ensuring reliability in medical information^13,14^.

While ChatGPT appears capable of delivering well-structured and balanced information regarding VTE, the lack of robust evidence-based justification raises important concerns. In high-risk clinical contexts such as thromboembolic prophylaxis, reliance on information that is not adequately supported by validated sources may lead to clinically inappropriate or potentially unsafe decisions.

The "moderate" classification within the TDKL framework reflects responses that are partially correct and understandable but lack essential clinical details such as dosage, duration, safety considerations, or evidence-based justification. Prior research has shown that AI systems can demonstrate moderate to high concordance with clinical guidelines; however, complete accuracy is rarely achieved. Reported concordance rates typically range between 55 and 80%^13,15^, which is consistent with our findings.

Previous studies have reported that ChatGPT is generally capable of generating recommendations broadly aligned with clinical guidelines for thromboembolic prophylaxis; however, important limitations remain. Notably, despite providing generally appropriate suggestions, ChatGPT has been shown to offer recommendations even in scenarios where current guidelines indicate insufficient evidence to support a clear clinical decision^16^. In addition, variability in recommendations, inadequate reflection of evidence strength, and the potential inclusion of unverifiable or non-existent references raise concerns regarding both reliability and clinical applicability^15^. Our findings further suggest that the accessibility of AI-generated information does not necessarily correspond to its accuracy or reliability. In high-risk clinical settings such as VTE, this limitation may contribute to clinically inadequate or potentially unsafe decision-making. This may partly explain why these models are primarily optimized for linguistic coherence rather than clinical accuracy. Furthermore, their limited ability to systematically interpret levels of evidence and strength of recommendations may result in incomplete or overly generalized outputs in certain clinical scenarios.

## CONCLUSION

This study suggests that although AI-generated information may exhibit high structural quality, it may not consistently achieve the same level of clinical adequacy. The observed discrepancy between DISCERN and TDKL highlights potential limitations of existing evaluation tools in capturing clinically relevant aspects of medical information.

These findings underscore the importance of developing clinically oriented assessment frameworks and suggest that AI-based systems should be used with caution in medical practice. AI-based systems may not be sufficient as standalone decision-support tools for high-risk clinical scenarios.

### Limitations

This study has several limitations. Only a single AI model was evaluated, analyses were restricted to guideline-based theoretical questions, and real-world clinical scenarios or patient outcomes were not assessed. However, the use of independent query sessions, high inter-rater agreement, and multiple evaluation instruments strengthens the methodological rigor of the study.

## Data Availability

The datasets generated and/or analyzed during the current study are available from the corresponding author upon reasonable request.

## References

[1] Falck-Ytter Y, Francis CW, Johanson NA, Curley C, Dahl OE, Schulman S (2012). Prevention of VTE in orthopedic surgery patients: Antithrombotic Therapy and Prevention of Thrombosis, 9th ed: American College of Chest Physicians Evidence-based clinical practice guidelines. Chest.

[2] Husted H, Otte KS, Kristensen BB, Ørsnes T, Wong C, Kehlet H (2010). Low risk of thromboembolic complications after fast-track hip and knee arthroplasty. Acta Orthop.

[3] Mont MA, Jacobs JJ, Boggio LN, Bozic KJ, Della Valle CJ, Goodman SB (2011). Preventing venous thromboembolic disease in patients undergoing elective hip and knee arthroplasty. J Am Acad Orthop Surg.

[4] National Guideline Centre (UK) (2018). Venous thromboembolism in over 16s: reducing the risk of hospital-acquired deep vein thrombosis or pulmonary embolism.

[5] The ICM-VTE Hip & Knee Delegates (2022). Recommendations from the ICM-VTE: hip & knee. J Bone Joint Surg Am.

[6] Uzel K, Azboy İ, Parvizi J (2023). Venous thromboembolism in orthopedic surgery: global guidelines. Acta Orthop Traumatol Turc.

[7] Magruder ML, Rodriguez AN, Wong JCJ, Erez O, Piuzzi NS, Scuderi GR (2024). Assessing ability for ChatGPT to answer total knee arthroplasty-related questions. J Arthroplasty.

[8] Yao JJ, Aggarwal M, Lopez RD, Namdari S (2024). Current concepts review: large language models in orthopaedics: definitions, uses, and limitations. J Bone Joint Surg Am.

[9] Celik H, Polat O, Ozcan C, Camur S, Kilinc BE, Uzun M (2020). Assessment of the quality and reliability of the ınformation on rotator cuff repair on YouTube. Orthop Traumatol Surg Res.

[10] Charnock D, Shepperd S, Needham G, Gann R (1999). DISCERN: an instrument for judging the quality of written consumer health information on treatment choices. J Epidemiol Community Health.

[11] Silberg WM, Lundberg GD, Musacchio RA (1997). Assessing, controlling, and assuring the quality of medical ınformation on the ınternet: caveant lector et viewor--let the reader and viewer beware. JAMA.

[12] Dünki A, Polat Ö (2026). Agreement between AI language models and BOOM chondrosarcoma consensus statements: a comparative study. Acta Orthop Belg.

[13] Gaudiani MA, Castle JP, Abbas MJ, Pratt BA, Myles MD, Moutzouros V (2024). ChatGPT-4 generates more accurate and complete responses to common patient questions about anterior cruciate ligament reconstruction than Google's search engine. Arthrosc Sports Med Rehabil.

[14] Kodra JD, Saroyan A, Darby F, Surucu S, Fong S, Gillinov S (2025). ChatGPT-generated responses across orthopaedic sports medicine surgery vary in accuracy, quality, and readability: a systematic review. Arthrosc Sports Med Rehabil.

[15] Megalla M, Hahn AK, Bauer JA, Windsor JT, Grace ZT, Gedman MA (2024). ChatGPT and Google provide mostly excellent or satisfactory responses to the most frequently asked patient questions related to rotator cuff repair. Arthrosc Sports Med Rehabil.

[16] Duey AH, Nietsch KS, Zaidat B, Ren R, Ndjonko LCM, Shrestha N (2023). Thromboembolic prophylaxis in spine surgery: an analysis of ChatGPT recommendations. Spine J.

